# Motivational Patterns as an Instrument for Predicting Performance Not Only in Football? A Replication Study With Young Talented Ice Hockey Players

**DOI:** 10.3389/fpsyg.2019.02357

**Published:** 2019-10-22

**Authors:** Claudia Zuber, Achim Conzelmann

**Affiliations:** Institute of Sport Science, University of Bern, Bern, Switzerland

**Keywords:** motivation, person-oriented approach, pattern analysis, performance, ice hockey

## Abstract

In football it was recently demonstrated, that patterns of motivational constructs in young talented football players are relatively stable in early adolescence, and are associated with specific performance related outcomes (Zuber et al., 2015). The aim of the present study was to check whether the motivational patterns found in youth elite football also re-emerge in ice hockey, showing similar relations to performance. 135 young male ice hockey talents (*M_Age_* = 17.26, *SD* = 1.24) playing on the highest and second highest level of their age group were questioned about five motivational constructs. Six months later, their coaches rated their players’ current game performance. The results demonstrated a very high similarity of the patterns in ice hockey with the ones earlier found in football. In terms of the transition to performance levels, the highly intrinsically achievement-oriented players were rated by their coaches as belonging to the top-level players significantly more often (*OR* = 2.6), whereas the non-achievement-oriented failure-fearing players show a higher chance to be rated as part of the lowest performance group (*OR* = 3.8). The results indicate the importance of achievement motivation for performance in ice hockey also, and this generalizability thus provides relevant clues, which are important for consideration in national programs for talent identification across several sports.

## Introduction

It is undisputed that achievement motivation is an essential puzzle piece for athletic success, which is why it is included in today’s multi-dimensional talent models (Morris, 2000). There are different theories subsumed under the umbrella term achievement motivation and those various constructs (Elliot and Dweck, 2007), to which importance is attributed for talent selection and talent development. Such theories are currently being intensively discussed as they show different prognostic relevance for success and performance in future (Murr et al., 2018). In order to do justice to the broad content of motivation, the implementation of a holistic approach (see Bergman and Magnusson, 1997) seems reasonable (Zuber et al., 2015). In doing so, motivation profiles - consisting of several constructs - are checked for their suitability as talent predictors. In such profiles, the interactions and possibilities of compensation between individual constructs, which is characteristic for talent development (Abbott et al., 2005), can be taken into account.^1^

The integration of holistic, developmental scientific concepts and their methodological consequences into the person-oriented approach has so far led to promising results for questions on talent selection (Zuber et al., 2015, 2016; Zibung et al., 2016) and talent development (Zibung and Conzelmann, 2013; Sieghartsleitner et al., 2018). The study of Zuber et al. (2015) examined the influence of the subsystem motivation on performance in a person-oriented manner. Based on Conroy et al. (2007), the motivational subsystem was formed by the frequently discussed motivational constructs of achievement motivation (Atkinson, 1957), achievement goal orientation (Duda, 1992) and self-determination (Ryan and Deci, 2000). Those three constructs have already been examined individually in connection with athletic performance in adolescence. A comprehensive example is the study with 2677 U12 football players in the German talent development program by Höner and Feichtinger (2016). They found that the achievement motives hope for success and fear of failure, as well as achievement goal orientation, demonstrated significant relationships with future success in the U16 age class. High self-determination was found to be connected with high performance levels in junior national fencers (Gillet et al., 2012). In a holistic way, these concepts have not been – to the best of our knowledge - investigated jointly, except for by Zuber et al. (2015). Their results showed that patterns of those motivational constructs in young talented football players are relatively stable over the age period of 12 to 14 years and are associated with specific performance related outcomes. Overall, four structurally stable patterns were found. The highly intrinsically achievement-oriented players showed high levels of win and goal orientation, hope for success, self-determination, and a low level of fear of failure. The win-oriented failure-fearing players were characterized by above-average levels of win orientation and fear of failure. The third pattern of the average motivated players showed – as the name suggests – no special features, whereas the non-achievement-oriented failure-fearing players were represented by low levels of the two achievement goal orientations, hope for success and self-determination and above-average pronounced fear of failure (c.f. Zuber et al., 2015). Relating to subsequent success, the highest probability to enter into the top-level of performance, namely the U15 national team, was found for the pattern of the highly intrinsically achievement-oriented players. In contrary, none of the non-achievement-oriented failure-fearing players succeeded to be nominated for the U15 national team. As an outlook of their results, Zuber et al. (2015) suggested to check in further longitudinal studies, “to what extent the identified clusters are also found in other sports and in other stages of development” (p. 8), and therefore if there is a generalizability of the subsystem itself to suggest prognostic relevance.

To work on this research gap, the aim of the present study was to check whether the motivational patterns found in youth elite football players also re-emerge in another age class in ice hockey, showing similar relations to performance. Ice hockey was chosen as it is one of the most popular sports in Switzerland, raising the importance of questions on talent identification and talent development. Nevertheless, the amount on talent research in ice hockey is very rare. To the best of our knowledge, no studies on achievement motivation and performance has be done to date. On the one hand, the present study aims to generate knowledge about the importance of achievement motivation in ice hockey. On the other hand, we value knowledge on the generalizability and reproducibility of results to other sports and age classes. We consider this interesting and important not only from a scientific point of view, but also from a practical point of view, being something to consider in national programs for talent identification (TID) across several sports (e.g., Fuchslocher et al., 2011). In addition, the present study can advance the state of research on the application of the person-oriented approach, and thus examine the quality of its usefulness for talent research.

## Materials and Methods

To check the generalizability of the results of Zuber et al. (2015), we chose a methodological approach as similar as possible to the original study: The examined motivational constructs, the measures used as well as the methods for data analysis have been replicated exactly. The present study differs in that (a) the motivational patterns were only assessed once and therefore no conclusion on individual stability can be drawn, (b) in the type of sport, (c) in the wider age and performance range of the participants, leading to a tougher test of the generalizability, and therefore – as there is no national team for every age class – (d) also in the performance criterion. Instead of the nomination to the national team, a coach evaluation on performance was used. As nominations for a national team are also a coach evaluation, there is no expected impact of this difference on the study results. Formal ethical approval was granted from the authors’ institutional review board before conducting the study and all players and their legal representatives provided their written informed consent to participate.

### Participants and Procedure

Between summer 2016 and 2018 135 male young ice hockey talents (*M_*Age*_* = 17.26, *SD* = 1.24) playing on the highest (*n* = 80; three teams out of two clubs) and second highest level (*n* = 55; three teams out of three clubs) of their age group were recruited for the study. The motivational variables were ascertained by means of questionnaires. Six months later, their coaches were asked to carry out the assessment of their players’ current game performance on a visual scale between 0 and 100 (Zuber and Conzelmann, 2014). In doing so, each player was compared nationally with the other players in his age class (*M_*Performance*_* = 60.89, *SD* = 16.82). As expected, the performance assessment differs on the two different levels (*M_*high*_* = 65.20, *SD*_*high*_ = 14.32; *M_*low*_* = 54.20, *SD_*low*_* = 18.30; *p* < 0.001), what speaks for the validity of the used method. Based on these ratings, the players were grouped into three performance levels similar in size (high level *n* = 46, *M* = 78.56, *SD* = 6.75; medium level *n* = 38, *M* = 60.16, *SD* = 3.28; low level *n* = 49, *M* = 42.62, *SD* = 10.56).^2^

### Measures

The German version of the short scale of the Achievement Motives Scale – Sport (AMS-Sport) (Wenhold et al., 2009) was used to measure the achievement motives hope for success (α = 0.80) and fear of failure (α = 0.82). The achievement goal orientations were measured using the scales win orientation (α = 0.80) and goal orientation (α = 0.71) of the German version of the Sport Orientation Questionnaire (SOQ) (Elbe, 2004). Self-determination was assessed using the Sport Motivation Scale (SMS) (Burtscher et al., 2011). The seven subscales were combined to form a self-determination index (Vallerand, 2001), where high, positive scores indicate a high level of self-determination (α = 0.86).

### Data Analysis

The LICUR method (cf. Bergman and El-Khouri, 2003) was used to analyze the subsystem motivation in a person-oriented way. All of the subsequently described statistical procedures were carried out using the statistical package ROPstat (Vargha et al., 2015). In the first step, extreme cases (residues) were identified and removed from the data set, since they would distort the cluster solution. This led to the exclusion of two cases with unique constellations of the referred constructs, as their Euclidean distance to each of the other cases exceeded the *T* = 0.8 threshold value for *z*-standardized data. For the subsequent cluster analysis, the Ward procedure with squared Euclidean distance was used. Afterward a partitioning cluster analysis (k-means method) was carried out to optimize the homogeneity within each cluster. Finally, the number of transitions from each cluster to the three performance levels was counted and tested for significance using Fisher’s exact test, with a hypergeometric distribution (*p* < 0.05). The amount of transitions was represented as a multiple of the expected value and expressed using odds ratios (*OR* = 1.0 as the expected value; *OR* < 1.0 means less and *OR* > 1.0 more transitions than expected by chance). To check for similarities between the clusters of the ice hockey players in the present study and the football players of the study from Zuber et al. (2015)^3^, the average squared Euclidean distance between the four pairs of most similar clusters of each sample was computed.

## Results

**FIGURE 1:**
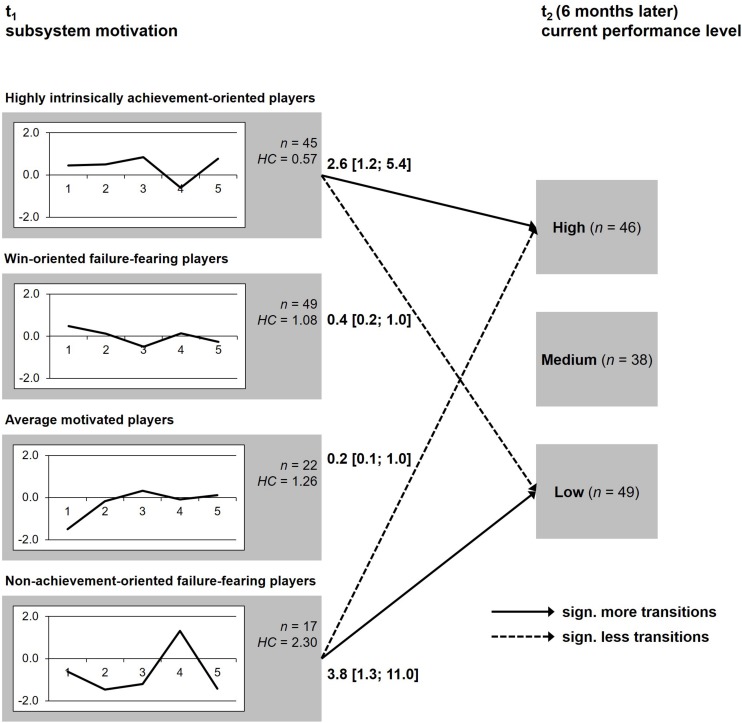
Profiles of *z*-scores of the four clusters and transitions to performance levels. Motivational constructs: 1, win orientation; 2, goal orientation; 3, hope for success; 4, fear of failure; 5, self-determination; numbers for significant more (OR > 1.0) or less (OR < 1.0) transitions are expressed as odds ratios and 95% confidence intervals.

Table 1 provides an overview of the descriptive statistics for the five motivational constructs of all the clusters and the overall sample. The cluster analysis extracted the supposed four-pattern solution (cf. Figure 1) and displayed with *EESS* = 46.9.1%; *CL*_*delta*_ = 0.88, weighted *HC*_*mean*_ = 1.09 and silhouette coefficient = 0.55 an acceptable pattern solution (Vargha et al., 2016). With the exception of cluster 4, which contains the lowest numbers of players, the homogeneity coefficients lie in an acceptable range. The comparison of the clusters from the football study (Zuber et al., 2015) and the present results show a very high similarity (*M_*d*_^2^* = 0.16), which is why they were named the same.

**TABLE 1:** Descriptive statistics for the subsystem motivation.

	**Overall (*n* = 133)**		**Highly intrinsically achievement-oriented players (*n* = 45)**		**Win-oriented failure-fearing players (*n* = 49)**		**Average motivated players (*n* = 22)**		**Non-achievement-oriented failure-fearing players (*n* = 17)**	
	***M***	***SD***	***M***	***SD***	***M***	***SD***	***M***	***SD***	***M***	***SD***
Win orientation (range 1–5)	4.32	0.63	4.60	0.36	4.62	0.30	3.38	0.42	3.93	0.70
Goal orientation (range 1–5)	4.59	0.39	4.79	0.26	4.64	0.28	4.52	0.33	4.02	0.46
Hope for success (range 0–3)	2.43	0.46	2.81	0.22	2.20	0.32	2.57	0.36	1.88	0.44
Fear of failure (range 0–3)	0.56	0.54	0.23	0.26	0.63	0.47	0.51	0.48	1.27	0.63
Self-determination (range −18–18)	8.13	3.01	10.46	1.33	7.33	2.55	8.45	2.24	3.85	2.67

From clusters 1 and 4, significant paths to the performance groups were revealed (see Figure 1): The highly intrinsically achievement-oriented players were rated by their coaches as belonging to the top-level group of players significantly more often (*OR* = 2.6) and to the lowest performance level (*OR* = 0.4) significantly less often. In contrary, the non-achievement-oriented failure-fearing players show a much lower chance to belong to the top-level players (*OR* = 0.2) and a higher chance to be rated as containing to the lowest performance group (*OR* = 3.8). The win-oriented failure-fearing and the average motivated players were rated as belonging to all three performance levels equally as often.

## Discussion

The current study investigated whether the motivational patterns found in youth elite football players (Zuber et al., 2015) re-emerge in ice hockey and show similar relations to performance. The present results are in line with the findings of Zuber et al. (2015) to a considerable degree. The patterns in both samples as well as the relations to the performance criteria show a very high similarity. Taken together, we interpret the results of this study as evidence that the structure of the four patterns on motivation as well as the prognostic relevance of the patterns show some degree of generalizability in the age range between 12 and 20 years in team sports in Switzerland. The assumption of generalizability is additionally exacerbated by the fact that the sample of hockey players is more heterogeneous in terms of the included performance level (not only top-level, but also second-highest-level) and a much wider age range than the football sample. As to the best of our knowledge, there is no empirical research in ice hockey on motivation and performance until today. Therefore, this is the first study giving insights into the importance of achievement motivation for performance in youth elite ice hockey players.

Unfortunately, it is hardly possible to incorporate these results into the current state of research. Besides two reviews on psychological factors, which are completely focused on football (Gledhill et al., 2017; Murr et al., 2018), only Johnston et al. (2018) systematically reviewed differences in performance variables from several domains between highly skilled and less-skilled athletes in sports generally. However, they only included one study dealing with motivational variables (in youth field hockey). Reviews regarding the significance of psychological characteristics for talent development in various sports are lacking, which is why findings on the generalizability for different sports are so important.

From a methodological point of view, it should be noted that cluster analytical methods (like other statistical methods) each have a certain arbitrariness or a high number of degrees of freedom. However, results which speak for the transferability of cluster solutions to other samples help to reduce this uncertainty and therefore point in our study to relatively stable and thus not coincidentally existing profiles (known as common types; Bergman and El-Khouri, 2003) which seem to fit optimally into the overall system of youth elite athletes in soccer and ice hockey.

In terms of practical implications, these results could help National Olympic Associations promoting general talent identification programs (TID) integrating all sports to derive valid recommendations, or develop instruments useable in different sports.

As in ice hockey there is no national team in every age class^4^, the chances for the nomination in a national team for the younger players of a 2-year cohort might be reduced, meaning that the coach ratings seemed to be the more valid performance criterion. But one of the possible weaknesses of the study is that the coach ratings show some kind of subjective bias. However, due to the time lag between the self-assessment of the athletes (and thus the request to the coaches for participation in the study) and the rating of the performance 6 months later, a systematic bias in the sense of a confirmation bias of the study goals seems unlikely. Furthermore, coaches’ ratings of the overall performance of players and overall potential show high inter-rater reliability (Zuber and Conzelmann, 2014; Fenner et al., 2016; Güllich et al., 2017).

Despite this limitation, the results of this study indicate the importance of achievement motivation for performance also in ice hockey and helped gain further knowledge of the generalizability of motivational patterns. Further studies should now check this generalizability also in individual sports and over a longitudinal period.

## Data Availability Statement

The datasets generated for this study are available on request to the corresponding author.

## Ethics Statement

This study was carried out in accordance with the recommendations of the ethics committee of the Philosophical-Humanistic Faculty at the University of Bern. All subjects gave written informed consent in accordance with the Declaration of Helsinki. The protocol was approved by the ethics committee of the Philosophical-Humanistic Faculty at the University of Bern.

## Author Contributions

CZ contributed to the conception or design of the work and the acquisition, analysis, and interpretation of data for the work; draft of the article; final approval of the version to be published; agrees to be accountable for all aspects of the work in ensuring that questions related to the accuracy or integrity of any part of the work are appropriately investigated and resolved. AC performed the interpretation of data for the work; critical revision of the drafts; final approval of the version to be published; agrees to be accountable for all aspects of the work in ensuring that questions related to the accuracy or integrity of any part of the work are appropriately investigated and resolved.

## Conflict of Interest

The authors declare that the research was conducted in the absence of any commercial or financial relationships that could be construed as a potential conflict of interest.

## Footnotes

1 It is comprehensively worked up in Zibung and Conzelmann (2013) how the person-oriented approach satisfies the integration of the dynamic-interactionist concepts of development in talent research.

2 Final *n* = 133, as two residues were removed (see section “Data Analysis”).

3 From the data of Zuber et al. (2015) the *z*-values from t_2_ were used, as the football players at that measurement point were more similar in age to the ice hockey players in the present study.

4 There is no U19 national team.
